# Supplementary material for: A UMLS-based spell checker for natural language processing in vaccine safety
Source: BMC Med Inform Decis Mak. 2007 Feb 12;7:3. doi: 10.1186/1472-6947-7-3 (PMC1805499; doi:10.1186/1472-6947-7-3)
Supplement: Additional file 13 — RAP application directory. Sets up directory for RAP (RDF application for PHP) source code [file 1472-6947-7-3-S13.gz › rap/doc/phpdoc/li_dataset.html]

xml version="1.0" encoding="iso-8859-1"?


dataset

Description
:   Class trees  
    Index of elements  
    Todo List

Classes
:   Dataset
:   DatasetDb
:   DatasetMem
:   IteratorAllGraphsDb
:   IteratorAllGraphsMem
:   IteratorFindQuadsDb
:   IteratorFindQuadsMem
:   NamedGraphDb
:   NamedGraphMem
:   Quad
:   TriXParser
:   TriXSerializer

Files
:   Dataset.php
:   DatasetDb.php
:   DatasetMem.php
:   IteratorAllGraphsDb.php
:   IteratorAllGraphsMem.php
:   IteratorFindQuadsDb.php
:   IteratorFindQuadsMem.php
:   NamedGraphDb.php
:   NamedGraphMem.php
:   Quad.php
:   TriXParser.php
:   TriXSerializer.php

phpDocumentor v 1.3.0RC3
